# Iron Oxide Nanoparticle-Assisted Delamination of Ti_3_C_2_T_x_ MXenes: A New Approach to Produce Magnetic MXene-Based Composites

**DOI:** 10.3390/nano14010097

**Published:** 2023-12-30

**Authors:** Kirill Sobolev, Alexander Omelyanchik, Nikolai Shilov, Mikhail Gorshenkov, Nikolai Andreev, Antonio Comite, Sawssen Slimani, Davide Peddis, Yevgeniy Ovchenkov, Alexander Vasiliev, Kurban E. Magomedov, Valeria Rodionova

**Affiliations:** 1 REC Smart Materials and Biomedical Applications, Immanuel Kant Baltic Federal University, A. Nevskogo Str. 14, 236014 Kaliningrad, Russia; aleksander.omelianchik@ext.unige.it (A.O.); kmagomedov@kantiana.ru (K.E.M.); 2 Department of Materials Engineering, Ben Gurion University of the Negev, P.O. Box 653, Beer-Sheva 8410501, Israel; 3 Department of Chemistry and Industrial Chemistry & INSTM RU, University of Genova, Via Dodecaneso 31, 16146 Genova, Italydavide.peddis@unige.it (D.P.); 4 Institute of Structure of Matter, National Research Council, nM^2^-Lab, Monterotondo Scalo, 00015 Rome, Italy; 5 National University of Science and Technology “MISiS”, Leninsky Pr. 4b1, 119049 Moscow, Russiaovtchenkov@mig.phys.msu.ru (Y.O.);; 6 Faculty of Physics, Lomonosov Moscow State University, Kolmogorova Str. 1/2, 119234 Moscow, Russia; 7 Faculty of Chemistry, Dagestan State University, M. Gadzhiev Str. 43-a, 367000 Makhachkala, Russia

**Keywords:** MXenes, magnetic nanoparticles, Ti_3_C_2_T_x_, Fe_3_O_4_, chemical delamination, composite materials, adsorption

## Abstract

Ti_3_C_2_T_x_ MXene is one of the most comprehensively studied 2D materials in terms of its adsorptive, transport, and catalytic properties, cytotoxic performance, etc. Still, conventional MXene synthesis approaches provide low single-flake MXene yield and frequently uncontrollable properties, demanding further post-processing. The MXene family also lacks magnetism, which is helpful for producing effective nanoadsorbents as their magnetic decantation is the cheapest and most convenient way to remove the spent adsorbent from water. Composite materials consisting of magnetic nanoparticles grown on top of MXene flakes are commonly used to provide magnetic properties to the resulting nanocomposite. In this paper, we study the possibility to delaminate multilayer Ti_3_C_2_T_x_ MXene sheets directly by growing iron oxide magnetic nanoparticles inside their interlayer spacing. We find out that, with a mass fraction of particles comparable or exceeding that of MXenes, their growth is accompanied by an effective enhancement of single-layer MXene yield and suitable magnetic properties of the resulting composite. The developed approach can be further used for simplifying synthesis protocols to obtain magnetic MXene-based nanoadsorbents with tunable properties.

## 1. Introduction

MXenes are the novel class of 2D transition metal carbides/nitrides and carbonitrides with M_n+1_X_n_T_x_ chemistry, where M is an early transition metal (Ti, Mo, V, etc.), X—C or/and N, and T_x_ is a surface functional group (most commonly –O, –OH and –F); n is an integer index, typically ranging from 1 to 4 [1]. MXenes were mentioned for the first time by Naguib, Gogotsi et al. in 2011 [2], after which this topic skyrocketed [3]. MXenes MXenes were found to be promising in such areas as energy storage, catalysis, wearable electronics, sensorics, environmental remediation, biomedicine, etc. [4]. MXenes inherit wide chemical and stoichiometric diversity, as well as high electrical conductivity, from their parental MAX phases [5,6]; naturally, they demonstrate large specific surface area with a large number of active sites, and an outstanding hydrophilicity. These properties ensure that MXenes are not only applicable in dozens of practical areas but, even more importantly, tunable for each specific application [4,7]. Typically, MXenes are prepared by a top-down approach via a selective etching of the nanolamellar MAX phase precursor using different reagents (HF, HCl + LiF, NaOH, KOH, molten salts, etc.) [2,8,9,10,11]. One major drawback is that each synthesis method does not provide the complete delamination of the parental MAX phase, so the resulting product consists of a mixture of multilayer (ML) MXene stacks, single-layer (SL) MXene flakes, and non-reacted MAX phase leftovers [12]. The use of different reagents also provides MXenes with various termination groups (T_x_) [12]. For the predominant part of the known synthesis techniques, ML MXene yield is much higher than that of SL flakes [12,13,14], which are much more promising as they possess better colloidal stability and larger specific surface area [10,15]. There are several methods to further delaminate ML MXenes: ultrasound exposure, metal ion or large organic molecule intercalation (DMSO or TMAOH are the most common choice for the latter case), etc. [8,16,17,18,19]. However, even these methods are frequently followed by further processing to obtain the material with the desired properties, i.e., delamination does not end up with the ready-to-use product [20,21].

MXene–nanoparticle (MXene-NP) composite materials are the novel, rapidly evolving branch of 2D materials science. NPs of different compositions, sizes, and shapes have been successfully grown on MXenes to provide the resulting composites with enhanced adsorptive, catalytic, plasmonic, or magnetic properties [22,23,24,25,26]. One promising field to apply MXene-NP composites is lithium (and beyond lithium) ion batteries. It was shown that such composites form porous conductive networks, which accumulate charge carrier ions with excellent capacity and outstanding cyclic stability [27,28,29,30,31]. At the same time, MXenes functionalized with magnetic NPs (MNPs) can be very helpful as nanoadsorbents for water remediation [22]. Owing to their large specific surface area and chemical reactivity, MXenes are intensively explored as adsorbing agents to remove heavy metal and radioactive ions, dyes, and pharmaceutical leftovers from the aqueous media [32,33,34,35,36]. Magnetic decantation is a cheap and effective approach to remove the adsorbent particles from water after the completion of the adsorption process, as it was previously shown by many works in the nanoparticles field [37,38,39]. As none of the experimentally produced MXenes have yet demonstrated strong ferro- or ferrimagnetism [40], MNPs, for example, iron oxide (Fe_3_O_4_) grown on top of 2D flakes, are promising for inducing magnetism in these nanoadsorbents [22]. However, the technology of growing MNPs on MXenes is still at its infancy, and a great deal of further work has to be carried out before these composite materials can be used in real life.

NPs in general have never been considered as a tool to delaminate ML MXenes directly at the synthesis stage. Different approaches to produce nanoparticles in the presence of MXene flakes were utilized, and in all of them MXenes acted as nucleation centers for NP growth. Among them, the co-precipitation technique is the most widespread [41,42,43]. The variation of synthesis conditions, i.e., the concentration of reagents, temperature, time, etc., allows one to prepare NPs of different sizes and shapes [44,45,46]. When MXenes come into play, NPs grow directly on their surface [26]. In such a way, particles with different sizes may also intercalate the ML MXene structure, spreading the sheets away and leading to their delamination. Moreover, in this case, the material can be practically used directly after synthesis. As mentioned earlier, SL MXene yield is much poorer compared to ML for the majority of the explored synthesis protocols, for example, HCl + LiF mild (minimally intensive layer delamination) etching [47]. The potential application in water remediation technologies demands a huge amount of the material, which reflects the necessity to qualitatively improve MXene production yield [48]. NP-assisted delamination can be a great tool to simultaneously gain the increment in SL MXene yield and to obtain materials with an intrinsic magnetism.

In this work, we grow Fe_3_O_4_ MNPs in a solution of ML Ti_3_C_2_T_x_ MXenes, collected from centrifugation sediment after commonly used mild HCl + LiF synthesis. We study how exactly the growth of MNPs in the solution affects the stacking between neighboring MXene sheets, thoroughly investigate the magnetic properties of the obtained nanocomposite materials, and show their applicability in water remediation technologies by studying their adsorption capacity towards Cu^2+^ in water. Cu is a toxic heavy metal, whose short- and long-term acute exposure causes food poisoning, gastrointestinal illness, nausea, and the disruption of homeostasis in the human liver [49,50,51,52,53,54,55]. According to the World Health Organization (WHO), an acceptable limit for Cu^2+^ in the drinking water is 1.5 mg/L [56], and its presence above the specified limit must be eliminated.

The results of this work provide an alternative pathway toward the large-scale production of functional MXene-based nanoadsorbents, enriched with magnetic properties.

## 2. Materials and Methods

### 2.1. Synthesis of MXenes

For the synthesis of Ti_3_C_2_T_x_ MXenes, the parental Ti_3_AlC_2_ MAX phase was firstly produced using a standard pressureless sintering technique, referring to works [57,58]. X-ray diffraction (XRD) analysis, scanning electron microscopy (SEM) and energy dispersive X-ray spectroscopy (EDS) eliminated the presence of Ti_3_AlC_2_ and TiC phases in the obtained MAX phase samples. Notably, the presence of TiC by-phase does not affect the delamination behavior that is discussed below. MXenes were synthesized by selectively etching Al from the precursor MAX phase [8,47]: 1 g of Ti_3_AlC_2_ (with a particle size < 45 μm) was dispersed into a 10 mL 9 M HCl solution with 1 g of LiF (Sigma Aldrich, St. Louis, MI, USA); the reaction took place at 40 °C for 24 h, and the etching was supplied with continuous magnetic stirring. After this, the MXene suspension was centrifuged 7 times at 3000 rpm for 5 min with a stepwise DI water rinsing to remove unneeded products and reach a neutral pH. Single-flake MXenes forming a stable colloidal suspension were removed from the sediment, containing multilayer MXene stacks (ML MXenes), the remains of the non-reacted MAX phase, and TiC by-phase. The sediment was collected for further investigation, for which it was dried on a glass plate at 60 °C for 24 h in air.

### 2.2. Synthesis of Iron Oxide Fe_3_O_4_ Nanoparticles

Iron oxide (Fe_3_O_4_) MNPs were prepared through the co-precipitation of iron salts in an aqueous solution by a base, as reported in our previous work with some modifications [45,46]. The yield was preliminary adjusted to match the weight quantity of MXenes (~100 mg). Classical agitation with a mechanical stirrer does not allow the mixing of a small volume of liquid, and the concentration of salts used in this experiment was much lower than what is commonly used in the literature. So, in addition to mechanical stirring, we performed an ultrasound-supported co-precipitation reaction to reduce the volume of the solution [59].

The following reagents were used for the preparation of MNPs in composites: iron (II) sulfuric acid 7-aqueous (FeSO_4_ × 7H_2_O, >98%, LenReactiv, Saint Petersburg, Russia), iron (III) chloride 6-aqueous (FeCl_3_ × 6H_2_O, >98%, LenReactiv, Saint Petersburg, Russia), and aqueous ammonia (NH_4_OH 30%, Sigma Tech, Khabarovsk, Russia). Deionized water was used during the entire process of solution preparation. All purchased materials were used as received.

In the mechanical mixing method, 1 mmol of iron sulfate and 2 mmol of iron chloride (Fe(II)/Fe(III) = 1/2) were dissolved in 100 mL of distilled water. The solution was then placed into a 200 mL two-neck flask and heated up to 80 °C. Then, an excess of aqueous ammonia was added to achieve an alkaline pH value of 11, with intensive stirring using a mechanical stirrer. After 2 h, the black precipitate was separated from the solution using a strong magnet. Samples produced with this method are hereafter referred to as ‘M samples’.

Co-precipitation under ultrasonic mixing was carried out similarly, but the same amount of iron salts was dissolved in 5 mL of distilled water. The reaction was conducted in a glass flask placed in an ultrasonic bath (60 W, 37 kHz) with a water temperature of 80 °C, and the reaction time was reduced to 1 h. Samples produced with this method are hereafter referred to as ‘US samples’.

In both cases, the dry powders for subsequent characterization were obtained by drying the sample in a drying oven at 80 °C for 12 h.

### 2.3. Synthesis of MXene-MNP Composite Materials

**Figure 1:**
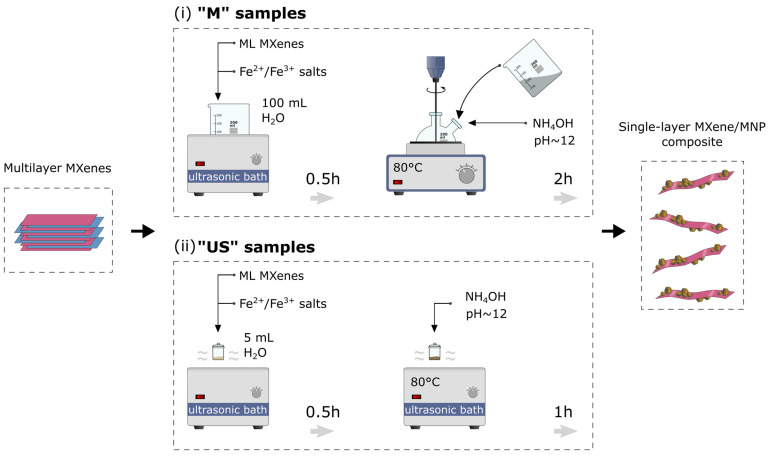
Schematic illustration of the MXene-MNP nanocomposite synthetic procedure, accompanied by the delamination of multilayer MXenes. The top panel shows the synthesis of “M” samples; the bottom panel shows the synthesis of “US” samples (see Table 1 for denotation).

Figure 1 schematically shows the synthesis procedure for both series of samples. All synthesis parameters are summarized in Table 1; the denotation of the obtained samples can also be found there.

#### 2.3.1. Co-Precipitation (M Samples)

Both approaches, mechanical intermixing and ultrasound assistance, were tested in the presence of a mild synthesis sediment, containing ML Ti_3_C_2_T_x_ MXene sheets. For the first approach, 1 mmol of ferrous sulfate with 2 mmol ferric chloride ([Fe^2+^]:[Fe^3+^] = 1:2) were dissolved into 100 mL distilled water, containing 100 mg of the sediment. Then, the temperature of the solution was brought up to 80 °C, and excessive ammonia solution was added under intensive stirring to achieve a highly basic medium. After 2 h, the black precipitate was collected with a strong NdFeB-based magnet. The magnetic decantation procedure was repeated several times with water and ethanol. Finally, the sample was dried in a drying oven at 80 °C overnight. 

#### 2.3.2. Ultrasound-Assisted Co-Precipitation (US Samples)

For the second approach, 1 mmol of ferrous sulfate with 2 mmol ferric chloride ([Fe^2+^]:[Fe^3+^] = 1:2) were dissolved into 5 mL distilled water containing various portions of the mild synthesis sediment, containing ML Ti_3_C_2_T_x_ MXene sheets (we used 50, 100, and 200 mg); the reaction was performed in an ultrasonic bath (60 W, 37 kHz) and the reaction time was reduced to 1 h. Three different concentrations of the sediment were chosen for the preparation in the ultrasonic bath as, due to the lower volume, synthesis there is more controllable, so the influence of the initial concentration on the resulting material can be checked. 

To summarize, for the ultrasound-assisted synthesis, the weight ratio between the MXene sediment and MNPs was 1:2, 1:1, and 2:1 (US:MX-50, US:MX:100, and US:MX-200 samples, respectively). For the mechanical agitation, the MXene/MNPs ratio was 1:2 (M:MX-100 sample).

### 2.4. Characterization of the Materials

We used XRD analysis and transmission electron microscopy (TEM) to structurally characterize all prepared samples. SQUID and vibrating sample magnetometry (VSM) were used to study their magnetic properties. XRD patterns were collected at room temperature using an AXRD Benchtop (Proto Mfg., Taylor, MI, USA) powder X-ray diffractometer with 30 kV and 30 mA monochromatic Cu-Kα radiation, with a step size of 0.015° and a counting time of 5 s per step at 2θ range of 5–80°, to check the phase composition of the samples and calculate the interlayer distances of the MXenes at each stage of the synthesis.

TEM investigations were carried out using a JEOL 1400 transmission electron microscope operating at 120 kV of the high tension. The samples for the TEM studies were prepared from a suspension by admixing a small amount of powder into 2 mL of ethanol. These suspensions were ultrasonically treated for 10 min, and a droplet of the suspension was placed onto a surface of a carbon-coated copper grid. After drying the grid in a vacuum desiccator, it was placed into the column of the microscope and then investigated in the bright-field and diffraction modes. 

We also performed dynamic light scattering (DLS) studies to extract information regarding the hydrodynamic sizes of MXene-MNP samples and to better understand the effect of NPs on stacked MXene structures. DLS measurements were performed using a Malvern Zetasizer Nano ZS equipped with a 633 nm red laser and operating at an angle of 90°. We used Zetasizer software version 7.2 from Malvern to collect and analyze the data. A total of 5 mg of each sample was dissolved into 5 mL of deionized water and mixed for 24 h. After that, 1 mL of the supernatant was measured using a polystyrene cuvette. The measurements were conducted at the fixed temperature of 25 °C; for each sample, 11 runs of 10 s each were performed, with 3 repetitions. The intensity size distribution and Z-average diameter (Z-ave) were obtained from the autocorrelation function. 

Physisorption measurements were carried out at 77 K using an ASAP 2020 MP Plus (Micromeritics, Norcross, GA, USA) instrument using N_2_ (full isotherm) and Kr (specific surface area via the Brunauer, Emmett, and Teller (BET) method). Samples were degassed at 180 °C prior to the analysis.

The magnetic properties of the MXene-MNP composites were firstly probed by means of the Lake Shore 7400 series (Cryotronics Inc., Westerville, OH, USA) vibrating sample magnetometer (VSM) at room temperature (~297 K), with the external field varying in the range of ±1.2 T. Powder samples were fixed in a tape container that was further attached to a quartz sample holder. By fitting the obtained hysteresis curves, the main magnetic parameters, i.e., saturation magnetization (*M_S_*), reduced remanent magnetization (*M_R_*/*M_S_*), and coercivity field (μ_0_*H_C_*), were calculated. For examining *M* vs. *T* behavior we used a Quantum Design Physical Properties Measurements System PPMS-9T (Quantum Design Inc., San Diego, CA, USA) in the range of 2–300 K. Zero-field-cooling (ZFC) and field-cooling (FC) magnetization measurements were performed at μ_0_*H* = 25 mT. The ZFC curve represents the magnetization behavior when the sample is cooled in the absence of an external magnetic field and then measured while applying a small field. On the other hand, the FC curve represents the magnetization behavior when the sample is cooled and measured while applying a constant magnetic field.

To demonstrate the efficiency of our materials for water remediation applications, we studied the adsorption of Cu^2+^ ions onto the US:MX-50 sample, possessing the most promising set of properties. For this, we used a stock solution of Cu^2+^ (15 mg/L) prepared by dissolving a specific amount of CuSO_4_·5H_2_O in bidistilled (BD) water at RT, and 10 mg/L BD water solutions of the US:MX-50. Standardized HCl and NaOH were used to control the pH of the solution in the range of 2.7 to 7.5; all measurements were performed at RT. The samples were analyzed using flame atomic absorbance spectroscopy novAA300 (AnalytikJena, Jena, Germany) at a wavelength of λ = 324.7 nm in order to determine the residual Cu^2+^ concentration. The absolute adsorption capacity at equilibrium was measured according to Equation (1):

(1) $$Q_{e}=\frac{{(C}_{0}–C_{e})*V}{m},\;$$

where *Q_e_* is the amount of Cu^2+^ adsorbed onto the US:MX-50 material at equilibrium, *C*_0_ and *C_e_* are the concentrations of Cu^2+^ at the initial point and at equilibrium, respectively, *V* is the volume of the solution, and *m* is the mass of the US:MX-50.

## 3. Results and Discussion

### 3.1. Characterization of MXene-MNP Composites

By means of an XRD analysis, we checked that magnetite MNPs synthesized using two different approaches (mechanical intermixing and ultrasound treatment) consist of a γ-Fe_2_O_3_/Fe_3_O_4_ spinel phase solely, with no impurity contamination; we also probed their magnetic properties using VSM. We found out that the minimization of the synthesis volume does not affect the resulting product. The ultrasound-assisted approach provides MNPs with sizes of 11–13 nm, compared to 7–9 nm for the mechanical stirring. The saturation magnetization (*M_S_*) of the US:Fe_3_O_4_ MNPs is about 73–79 A m^2^ kg^−1^, which is close to the value of the bulk Fe_3_O_4_ [60], indicating their excellent crystallinity. For the M:Fe_3_O_4_ MNPs, the *M_S_* value is almost two times lower, namely, 39 ± 3 A m^2^ kg^−1^, which may be attributed to the formation of the amorphous iron oxide phase when mechanical intermixing is used; this effect was previously described in the literature [45,61]. The detailed characterization of MNPs is given elsewhere [59].

**Figure 2:**
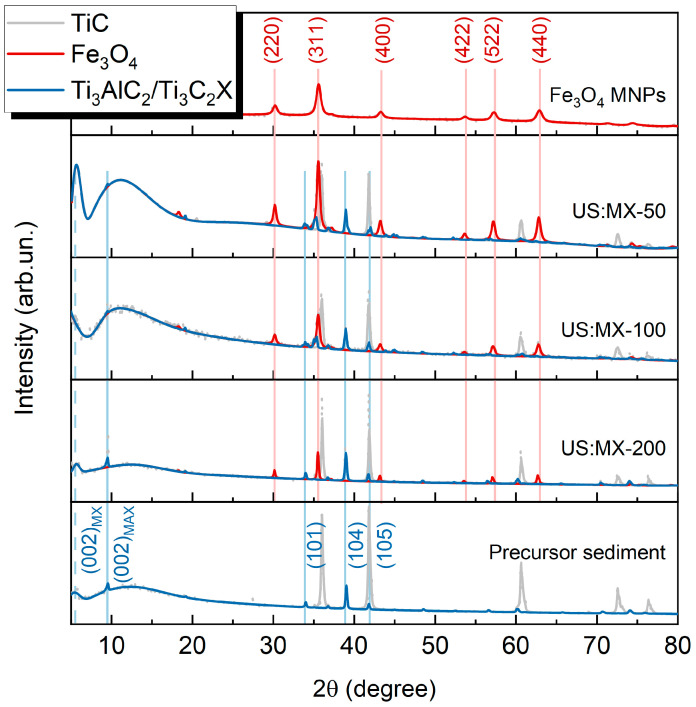
XRD patterns of MXene-MNP composite materials, synthesized according to the ultrasound treatment protocol: US:MX-50, US:MX-100, and US:MX-200; red curves represent Fe_3_O_4_ MNP reflections while the blue ones refer to Ti_3_C_2_T_x_ MXenes and the Ti_3_AlC_2_ MAX phase. The patterns for MNPs and the precursor MXene sediment are also shown. Gray curves indicate the TiC by-phase from the precursor.

The XRD patterns of MXene-based magnetic composites from the “US” series are given in Figure 2. The weight contents of Fe_3_O_4_, Ti_3_C_2_T_x_, and TiC phases are reported in Table 2. Changes in the mutual intensity of reflections, referring to MXenes and MNPs (blue and red curves, respectively), clearly follow the trend in their mass ratio. At the same time, the MXene delamination signature, i.e., the appearance of the broad peak at roughly 6°, becomes more and more pronounced with the increase in the relative concentration of MNPs. The position of the peak corresponds to an interlayer spacing of about 1.5 nm (except for the US:MX-100 sample where the peak demonstrates a larger left shift for reasons currently unclear). The pattern of the US:MX-200 sample with the highest MXene content has only minor traces of delamination. However, when the relative amount of MXenes decreases with respect to MNPs, the situation changes. The peak at 6°, although remaining at the same position, becomes more intense compared to (002) and (104) MAX phase reflections. Also, a wide reflection appears in the area around 10°. Notably, no extra peaks that could be assigned to the TiO_2_ phase appear on the scans, leading us to assume that our protocol does not lead to MXene oxidation.

The obtained results suggest that in the US:MX-100 and US:MX-50 samples, the yield of delaminated MXenes is much higher than it was in the initial precursor sediment, or in the US:MX-200 sample. The possible cause of this effect is the influence of iron salts, intercalating stacked MXenes flakes and providing further delamination, as was addressed earlier [62]. Positively charged Fe^2+^ and Fe^3+^ ions are electrostatically attracted to negatively charged Ti_3_C_2_T_x_ MXene flakes, and then a co-precipitation reaction takes place when iron bonds to oxygen and the total charge of the particle seed becomes neutral. Nanoparticle growth starts inside the ML structure, thus separating MXene sheets further from each other. As a result, MXene flakes with MNPs nucleating on their surface become more mobile.

**Figure 3:**
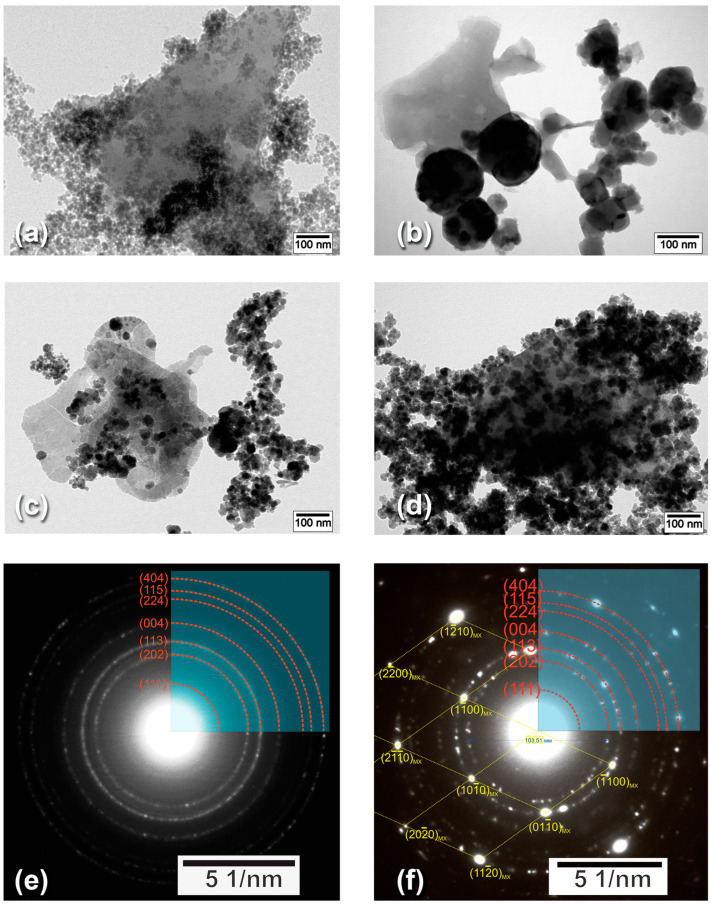
TEM images of the (**a**) M:MX-100, (**b**) US:MX-200, (**c**) US:MX-100, and (**d**) US:MX-50 samples. Electron diffraction patterns for (**e**) Fe_3_O_4_ MNPs solely and (**f**) Fe_3_O_4_ MNPs with Ti_3_C_2_T_x_ MXenes (the US:MX-50 sample).

To observe the addressed difference in a more evident way, we performed TEM imaging and analysis, the results of which are given in Figure 3. All bright-field images (Figure 3a–d) demonstrate single- or few-layer MXene flakes, which are irregular shape grey sheets, covered or surrounded by dark Fe_3_O_4_ particles of different sizes. The lateral sizes of the MXenes are about several hundred nanometers. 

For the M:MX-100 sample (Figure 3a), we can see the large amount of relatively small and uniformly sized MNPs, covering the entire surface of the Ti_3_C_2_T_x_ flake. Interestingly, for the US:MX-200 sample (Figure 3b), not only are the particles located exclusively at the interior of the MXene, but their size is much larger than what was discussed in the beginning of this section. For the US:MX-100 sample (Figure 3c), MNPs are obviously smaller and cover the inner part of the flake too. Finally, for the US:MX-50 sample (Figure 3d), where the weight ratio between the MXene sediment and the MNPs was 1:2 (just like in the case of the M:MX-100 sample), we see again a large amount of smaller particles uniformly covering the flake. In Figure 3e,f, the electron diffraction analysis patterns of the samples with and without MXenes, respectively, are given. Figure 3e demonstrates the electron diffraction pattern which is formed by randomly oriented Fe_3_O_4_ particles over the surface of the amorphous carbon film supporting the particles on the copper grid. The diffraction rings are marked by red lines, and the indexes of diffracting planes are provided to each ring. Figure 3f reveals the selected area diffraction pattern, consisting of two superimposed patterns—the first is the ring pattern from randomly oriented Fe_3_O_4_ particles (like in Figure 3e), and the second one is a single crystal diffraction pattern, which contains an array of diffraction spots from different atomic planes (the spots are marked by yellow lines to show their arrangement). The second pattern has a 6-fold symmetry, which is typical for MXene sheets; however, cubic Fe_3_O_4_ could also demonstrate a similar pattern if it has a sharp texture along the <111> axis. For that reason, the assignment between these spots and MXenes was confirmed by dark-field observation (dark-field images are not shown here), where the sheets were bright when the observation was performed using MXene spot reflection. 

Interestingly, TEM data suggest better crystallinity for the Fe_3_O_4_ phase in the US:MX-50 sample, compared to separately synthesized MNPs. This is in line with earlier assumptions from XRD data discussion, stating that the growth of Fe_3_O_4_ on the surface of Ti_3_C_2_T_x_ flakes provides better crystalline nanoparticles [59]. TEM also proves that MXenes do not oxidize during the described synthesis procedure, as we do not see any characteristic TiO_2_ particles on the flakes or TiO_2_-related features on the diffraction patterns. We additionally checked our samples using SEM-EDS (using TM4000II Tabletop SEM by Hitachi Ltd., Japan, with the QUANTAX 75 EDS detector by Bruker, Billerica, MA, USA), observing the absence of Al, and the presence of Fe and O, which supports previously given results.

**Figure 4:**
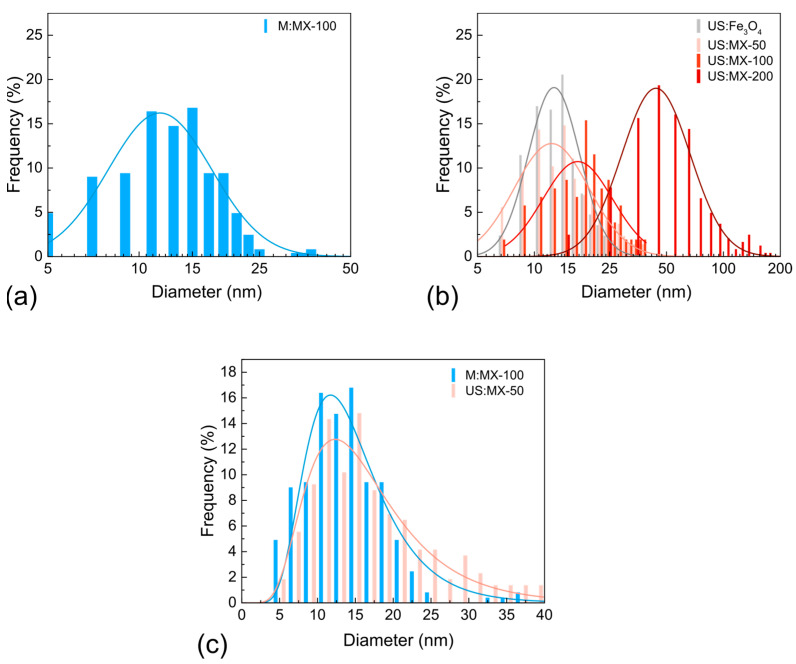
Particle size distributions for Fe_3_O_4_ MNPs from the (**a**) M:MX-100, (**b**) US:MX-200, US:MX-100, and US:MX-50 samples, calculated from the given TEM data; (**c**) demonstrates the comparison between MNPs size distributions in the M:MX-100 and US:MX-50 samples.

To examine the observed fascinating effect in more detail, we performed TEM image analysis using ImageJ software 1.51j8 [63]. The resulting size distributions of MNPs from different samples are given in Figure 4. The extracted values, together with XRD-based calculations (particle sizes and lattice parameters), are summarized in Table 2. In the “US” series, the size of MNPs increases monotonously from 15 nm for the US:MX-50 sample (almost the same as it was in the absence of MXenes) to 20 nm for the US:MX-100 sample, and to 52 nm for the US:MX-200 one. Following the discussion of the XRD results (see Figure 2), which addresses the delamination of MXenes when more iron salts are added to the system, this observation can be interpreted as follows. ML MXene stacks act as MNP nucleation centers. When the concentration of iron salts is relatively small, their intercalation rate is insufficient and, as a consequence, the delamination rate is also slow. As new nucleation sites do not appear, large Fe_3_O_4_ pieces grow directly on the surface of ML MXene particles. When the concentration of iron salts increases, the associated delamination provides more sites for MNP growth, so the average size of the particles becomes smaller and they tend to appear on the inner part of the flakes. This hypothesis is supported by the fact that for the samples with a more pronounced delamination, the MNP size distribution becomes wider. This may happen because MNPs do not start growing simultaneously but when more vacant nucleation sites become available. One can also note the deviation of the size, calculated using TEM and XRD, for the largest MNPs (e.g., in the US:MX-100 and US:MX-200 samples). This means that large MNPs are polycrystalline, unlike the small ones, so the Scherrer equation underestimates their size. Notably, the sizes and size distributions of MNPs from the M:MX-100 and US:MX-50 samples are very close to each other (see Figure 4c), which explains the similarity between these two samples, seen on TEM images.

To further support our findings, we performed DLS analysis, which revealed a general tendency of increasing hydrodynamic size with decreasing MNP content in agreement with the TEM analysis; the summary is given in Table 3. 

An increased amount of MXenes in the Fe_3_O_4_-MXene composite can potentially lead to an increase in the hydrodynamic size. As was addressed earlier, a larger MXene content causes MNPs of a bigger size to grow on the exterior of ML Ti_3_C_2_T_x_ particles. In fact, a higher MXene concentration can also hinder the dispersibility of Fe_3_O_4_ NPs in the composite, thus creating a greater likelihood of the nanoparticles aggregating or clustering together, which leads to larger hydrodynamic sizes as multiple particles are considered as a single entity. On the other hand, the observed broadening of the full width at half maximum (FWHM) values suggests a certain degree of size heterogeneity within the samples. This variation in size can be attributed to an unequal growth rate of MNPs on the exterior of ML MXene particles. 

All three samples from the “US” series showed a type II N_2_ physisorption isotherm with very little pronounced hysteresis. The shape of the isotherm reveals the non-porous or macroporous nature of the material [64]. The BET specific surface area (SSA_BET_) was determined using Krypton at 77 K, and the SSA_BET_ values decrease from about 34 m^2^/g to about 8 m^2^/g by increasing the MXene/MNP ratio (see Table 3). The results are consistent with the previous discussion since the amount of SL MXene flakes is assumed to be the largest for the US:MX-50 sample with the lowest MXene content.

### 3.2. Magnetic Properties

**Figure 5:**
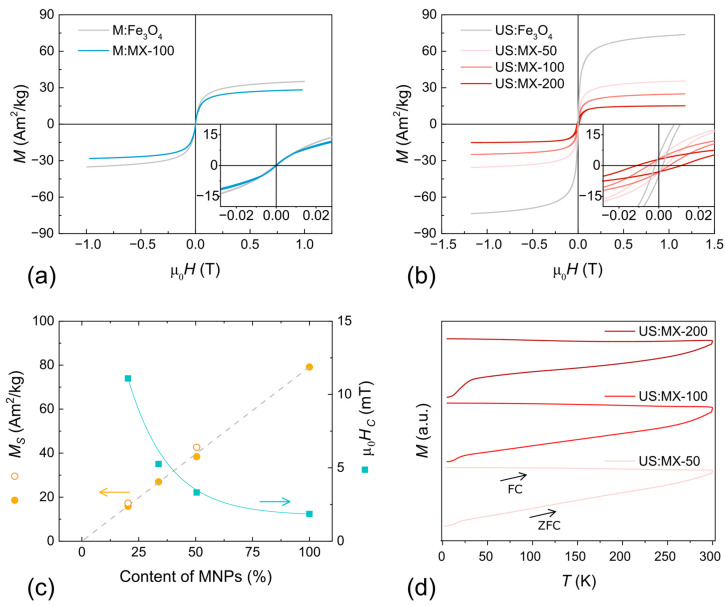
Magnetization versus applied field of MNPs and MXene-MNP composites prepared using (**a**) co-precipitation and (**b**) ultrasound-assisted co-precipitation. Insets show low-field regions. (**c**) Saturation magnetization and coercive field measured at 300 K for MNPs and MXene-MNP composites prepared using ultrasound-assisted co-precipitation as a function of the magnetic particle content (solid symbols by VSM; empty symbols by PPMS); (**d**) ZFC-FC magnetization recorded at μ_0_*H* = 25 mT.

The field dependences of magnetization (*M* vs. *H*) for M:Fe_3_O_4_ and US:Fe_3_O_4_ MNPs, and MXene-MNP composites, measured at RT, are given in Figure 5a,b. Iron oxide MNPs prepared using classical co-precipitation with mechanical agitation (Figure 5a) exhibit superparamagnetic behavior with almost vanishing remanence and coercivity. *M_S_* is relatively low (compared to the bulk magnetite or maghemite) and equal to 39 A m^2^ kg^−1^, which was attributed to the presence of an amorphous phase in the samples [59]. The magnetic properties of MNPs fabricated under the same conditions in the presence of 100 mg of Ti_3_C_2_T_x_ MXene-containing sediment also show superparamagnetic behavior. Comparing the magnetic properties of the M:Fe_3_O_4_ and M:MX-100 samples, one can notice that *M_S_* decreases slightly to 30 A m^2^ kg^−1^ in its absolute value. However, taking into account the mass ratio of non-magnetic MXenes in this sample, and normalizing the magnetization by the mass of MNPs, the *M_S_* value increases up to ~45 A m^2^ kg^−1^. In our case, the concentration of iron salts is low (0.03 mol L^−1^) compared to the classical co-precipitation, and a part of the material is in an amorphous state. As was mentioned earlier, MХenes act as crystallization centers in the co-precipitation process, improving the crystallinity, and, as a result, the saturation magnetization of the particles also increases. 

The ultrasound-assisted co-precipitation allows the performance of a reaction in a smaller volume of water, thus increasing the concentration of iron salts (0.15 mol L^−1^). As was also discussed earlier, iron oxide MNPs produced using this method possess a saturation magnetization of about 79 A m^2^ kg^−1^ (Figure 5b), which is close to the bulk value of the magnetic iron oxide, confirming the high crystallinity of the particles. In this approach, as the initial MNPs did not possess large amorphous parts, the addition of MXenes predictably decreased the M_S_ value of the resulting composites (Figure 5b). The calculated *M_S_* values for the MXene-MNP composites from the “US” series perfectly agree with what was expected from their mass ratio.

According to the observed behavior, coercivity and remanence measured at RT for the samples from the “US” series are larger than for the M:MX-100 sample. Moreover, the coercive field clearly increases in line from the US:MX-50 to the US:MX-200 sample (see the insert in Figure 5b). Figure 5c shows the calculated saturation magnetization and coercive field values as the function of the nominal content of nanoparticles in the samples. When the concentration of MXenes increases, coercivity also rises from 1.9 mT for pure MNPs up to 11.1 mT for the sample with the maximum MXene content (US:MX-200). This phenomenon can be associated with the increase in the particle size, observed using XRD and TEM, and discussed earlier in 3.1. The main magnetic parameters (*M_S_*, *M_R_*/*M_S_*_,_ and μ_0_*H_C_*) for the given MNPs and MXene-MNP composites are listed in Table 4. 

Figure 5d shows the ZFC-FC magnetization curves recorded at 25 mT. The ZFC-FC curves for all samples prepared using the ultrasound-assisted method exhibit a similar trend, where both ZFC and FC magnetization tend to merge at temperatures above RT. This observation agrees with the small but measurable residual magnetization and coercivity observed for these samples. This magnetic behavior could be advantageous for certain applications, such as water remediation or electromagnetic shielding [22,65], where a controlled and tunable magnetic response is desired.

**Figure 6:**
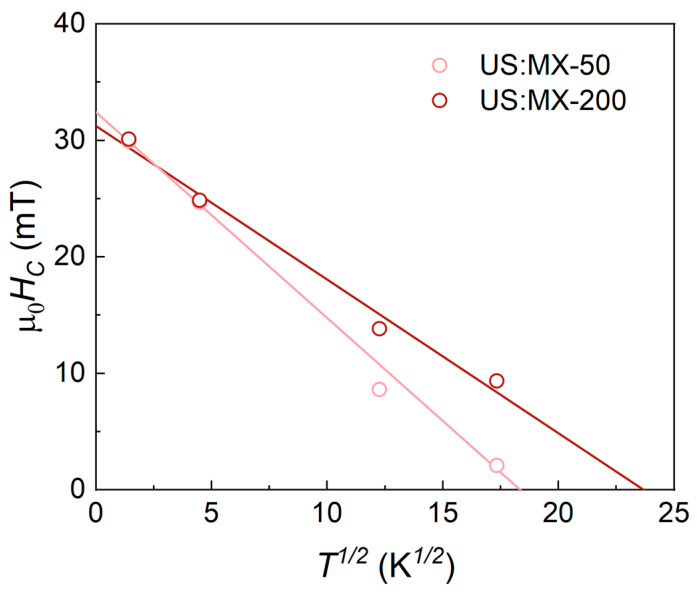
Temperature dependence of coercive field measured for US:MX-50 and US:MX-200 MXene-MNP composites prepared using ultrasound-assisted co-precipitation.

For the samples with the largest and smallest particles obtained using the ultrasound-assisted method (US:MX-200 and US:MX-50, respectively), we also measured hysteresis cycles at different temperatures. At very low temperatures (~2 K), the coercivities of both samples are almost identical. This can be explained by the increased contribution of surface anisotropy in smaller particles, which contributes to the total anisotropy. At low temperatures, the surface anisotropy increases due to disordered atoms on the surface, leading to higher coercivity [45,66]. At higher temperatures, the larger particles possess higher coercivity. In fact, as the temperature increases, thermal energies begin to approach and exceed the anisotropy barrier heights [67]. For the system with a larger average particle size (US:MX-200), a significant increase in temperature is required before thermal energies overcome the anisotropy barrier. However, for the system with smaller particles (US:MX-50), the anisotropy barriers are lower, and the additional contribution from the surface disappears. The thermal dependence of μ_0_*H_C_* was fitted with the rule μ_0_*H_C_*(T) = μ_0_*H_C_*(0)(1 − (*T*/*T_B_*)^1/2^), where *T_B_* is the blocking temperature, and μ_0_*H_C_*(0) is the coercivity at 0 K [68]; the result is given in Figure 6. For the US:MX-200 sample, the blocking temperature (*T_B_* = 560 ± 60 K) is larger than for the US:MX-50 one (*T_B_* = 336 ± 30 K). This is consistent with the larger volume of the US:MX-200 sample.

From the obtained results, we can conclude that the M:MX-100 and US:MX-50 samples, obtained using two different co-precipitation approaches, are the most promising ones for future studies and possible practical applications. Both samples demonstrate a significant nanoparticle-assisted delamination of the initial ML MXene particles.

### 3.3. Adsorptive Properties of MXene-MNP Composites

**Figure 7:**
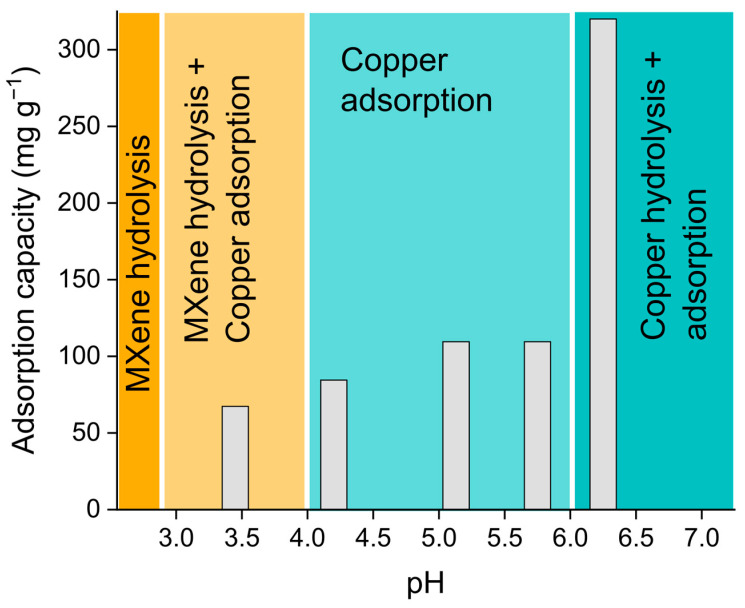
pH dependence of the absolute adsorption capacity of the US-50 MXene-MNP composite material prepared using ultrasound-assisted co-precipitation.

To estimate the adsorptive performance of the MXene-MNP composite material, we checked the adsorption capacity of the US:MX-50 sample towards Cu^2+^ ions as a function of the pH in the aqueous media. The pH range of 2.7–7.5 was screened; the result of this test is given in Figure 7. The whole pH range is visually divided into four different regions. In highly acidic environments (pH < 3, red area on Figure 7), adsorption equals to zero as MXene hydrolysis takes place, which agrees well with previous studies [60]. After that, at pH 3.0–3.5 (yellow area on Figure 7), the adsorption capacity rapidly increases, but still not reaching its maximum value. In this range, MXene hydrolysis and the protonation of its functional groups coexist with Cu^2+^ adsorption. At pH > 6.0 (blue area on Figure 7), copper hydrolysis will appear, and the copper hydroxide precipitate will form, so no adsorption on MXenes will take place. The most optimal adsorptive performance is observed in the pH range close to neutral, i.e., 4.0–5.5 (green area in Figure 7), and the maximum adsorption capacity is 105.8 mg/g at pH = 5. Notably, this value is almost identical to the highest one, which was previously reported for single-flake Ti_3_C_2_T_x_ MXenes—104 mg/g [49] (96 mg/g in another example [69]). 

The adsorptive behavior of the US:MX-50 sample being similar to single MXene flakes from [49] also supports the suggestion that MNP growth leads to MXene delamination, as the specific surface area is one of the most crucial factors determining the adsorptive properties of this material. The observed closeness of the capacities highlights that combining MXenes with MNPs does not reduce the adsorption capacity of the former, but supplies additional functionality related to the magnetic properties of the latter. The demonstrated behavior close to the superparamagnetic one allows the performance of magnetic decantation to extract the waste nanoadsorbent from the purified media. This approach is much easier and cheaper than filtration; moreover, magnetic decantation allows the collection of a large fraction of the adsorbent for remediation and further re-usage [37,38,39]. All these factors open the prospects for the large-scale industrial application and commercialization of the described technology.

With the evidence provided, we can conclude that the proposed method of delamination, mediated through iron salts, and leading to the simultaneous formation of iron oxide MNPs on the surface of MXene flakes, is successful and allows us to skip at least one preparation step when synthesizing magnetic MXene-based composites. Future research will focus on three main directions: (i) the adjustment of the MNPs’ chemical composition to finely tune the delamination rate and the resulting magnetic properties; (ii) the development of remediation protocols for waste nanoadsorbents to make them reusable; and (iii) the upscaling of the technology to check its capacity of producing 10^2^–10^3^ g batches in one cycle. All these directions are crucial for the commercialization of the technology, which we believe is possible due to the promising combination of the adsorptive and magnetic properties of the created MXene-based nanocomposite material.

## 4. Conclusions

We successfully prepared magnetic nanocomposite materials of Ti_3_C_2_T_x_ MXenes and Fe_3_O_4_ MNPs. When ML Ti_3_C_2_T_x_ MXenes were added to the precursor solution during the co-precipitation process of magnetite MNP growth, the concentration-dependent delamination of the former was observed, resulting in the significant enhancement of SL MXene yield. The iron salt intercalation of the ML Ti_3_C_2_T_x_ structure led to the spread of the neighboring sheets, which resulted in the appearance of vacant nucleation sites for further MNP growth. Thus, the size of the resulting MNPs became smaller with the reduction of MXene content. Due to this, the magnetic parameters, i.e., *M_S_*, *M_R_*/*M_S_*, and μ_0_*H_C_*, of the produced composites can also be finely tuned by the mutual concentration of reagents from almost superparamagnetic to more bulky. When the amount of MNPs exceeded that of the initial MXenes, the sample possessed an excellent delamination rate and magnetic properties suitable for the proposed application: the magnetic-field-mediated removal of MXene-based nanoadsorbents from water. In the performed adsorption study, the MXene-MNP nanocomposites demonstrated a significant Cu^2+^ adsorption capacity of 105.8 mg/g. The developed protocol provides an easy way to synthesize magnetic MXene-MNP composites, and can be successfully used for nanoadsorbent preparation.

## Figures and Tables

**Table 1:** Parameters used in the co-precipitation-based synthesis of Fe_3_O_4_ MNPs, and denotation of the obtained samples and series.

Sample	Agitation Type	Volume of Water, mL	Fe^2+^, mmol	Fe^3+^, mmol	Temperature, °C
M:Fe_3_O_4_	mechanical	100	1	2	80
M:MX-100	mechanical	100	1	2	80
US:Fe_3_O_4_	ultrasonic	5	0.25	0.5	80
US:MX-50	ultrasonic	5	0.25	0.5	80
US:MX-100	ultrasonic	5	0.25	0.5	80
US:MX-200	ultrasonic	5	0.25	0.5	80

**Table 2:** Structural properties of MNPs in the obtained samples: mean particle size (*d_TEM_*), standard deviation (*σ*), mean crystal size (*D_XRD_*), Fe_3_O_4_ lattice constant (*a*), as well as weight content of the Fe_3_O_4_, TiC, and Ti_3_C_2_T_x_ phases.

Sample	*d_TEM_* [nm]	*σ*	*D_XRD_* [nm]	*a* [Å]	Fe_3_O_4_ [%]	TiC [%]	Ti_3_C_2_T_x_ [%]
M:Fe_3_O_4_	—	—	7.5 ± 0.8	8.385 ± 0.007	100	—	—
M:MX-100	14	0.39	—	—	—	—	—
US:Fe_3_O_4_	14	0.31	12 ± 1	8.375 ± 0.008	100	—	—
US:MX-50	15	0.45	14 ± 1	8.377 ± 0.004	50 ± 5	32 ± 5	18 ± 3
US:MX-100	20	0.42	15 ± 3	8.351 ± 0.006	33 ± 4	45 ± 5	22 ± 3
US:MX-200	52	0.42	27 ± 5	8.378 ± 0.003	22 ± 3	50 ± 5	28 ± 4

**Table 3:** Hydrodynamic sizes (*d_h_*) and FWHM values of MXene and MNP composites produced using the ultrasound-assisted method, and specific surface areas calculated using the BET method (SSA_BET_) with Krypton as the sorbate.

Sample	*d_h_* [nm]	FWHM	SSA_BET_ [m^2^/g]
US:MX-50	209	25	34.2
US:MX-100	286	28	18.7
US:MX-200	447	162	7.9

**Table 4:** Magnetic properties of MNPs and MXene-MNP composite samples at 297 K: *M_S_* is the saturation magnetization, *M_R_*/*M_S_* is the reduced remanence, and μ_0_*H_C_* is the coercive field.

Sample	*M_S_* [A m^2^ kg^−1^]	*M_R_*/*M_S_*	µ_0_*H_C_* [mT]
M:Fe_3_O_4_	39 ± 3	—	—
M:MX-100	30 ± 3	—	—
US:Fe_3_O_4_	79 ± 4	0.06	1.9 ± 0.1
US:MX-50	38 ± 3	0.08	3.3 ± 0.1
US:MX-100	27 ± 3	0.12	5.3 ± 0.1
US:MX-200	16 ± 2	0.19	11.1 ± 0.1

## Data Availability

Data are contained within the article.
